# Longitudinal changes in HIV-specific IFN-γ secretion in subjects who received Remune™ vaccination prior to treatment interruption

**DOI:** 10.1186/1476-8518-4-7

**Published:** 2006-11-28

**Authors:** Kenneth H Huang, Marie-Pierre Boisvert, Famane Chung, Maude Loignon, Don Zarowny, Lise Cyr, Emil Toma, Nicole F Bernard

**Affiliations:** 1 McGill University Health Centre, Montreal, Quebec, Canada; 2 Centre hospitalier de l'Université de Montreal, Montreal, Quebec, Canada; 3 Canadian HIV Trials Network, Vancouver, British Colombia, Canada

## Abstract

**Background:**

Despite the benefits of highly active antitretroviral therapy (HAART) for suppressing viral replication in HIV infection, virus persists and rebounds during treatment interruption (TI). This study explored whether HAART intensification with Remune™ vaccination before TI can boost HIV-1-specific immunity, leading to improved control of viremia off HAART.

**Methods:**

Ten chronically HIV-infected adults were enrolled in this proof of concept study. After a 6-month HAART intensification phase with didanosine, hydroxyurea, granulocyte-macrophage colony-stimulating factor, (GM-CSF), and a first dose of Remune™ (HIV-1 Immunogen), HAART was discontinued. Patients continued to receive Remune™ every 3 months until the end of study. HAART was restarted if viral load did not fall below 50,000 copies/ml of plasma within 3 months or if CD4+ counts decreased to <200 cells/mm^3^. HIV-specific immunity was monitored with the interferon-γ (IFN-γ) ELISPOT assay.

**Results:**

All subjects experienced viral rebound during TIs. Although the magnitude and breadth of HIV-specific responses to HLA-restricted optimal peptide panels and Gag p55 peptide pools increased and viral load decreased by 0.44 log_10 _units from TI#1 to TI#2, no significant correlations between these parameters were observed. The patients spent 50.4% of their 36 months follow up off HAART.

**Conclusion:**

Stopping HAART in this vaccinated population induced immune responses that persisted after therapy was restarted. Induction of HIV-specific immunity beyond IFN-γ secretion may be contributing to better control of viremia during subsequent TIs allowing for long periods off HAART.

## Background

The introduction of highly active antiretroviral therapy (HAART) to the management of patients infected with HIV has significantly decreased mortality and morbidity [1]. Although HAART suppresses HIV replication in a significant proportion of HIV infected individuals, it is not able to eradicate viral infection [2,3]. Serious side effects and emergence of drug resistant virus provide the impetus to explore alternatives to continuous HAART [4,5].

HIV specific CD8+ T cells contribute to the control of HIV replication. The strongest evidence supporting this comes from an animal model of HIV infection, macaques infected with the simian immunodeficiency virus (SIV). In SIV infected macaques CD8+ T cell depletion results in increased viral load, which returns to pre-treatment values when CD8+ T cells reemerge [6]. Several other observations support a role for CD8+ T cells in control of HIV. These include viral escape from the CTL responses [7-10], the temporal association between decline in viral load and the emergence of CTL responses in HIV primary infection (PI) [11,12], the association of certain major histocompatibility complex (MHC) class I alleles and heterozygosity at loci coding for these alleles with rate of HIV disease progression [13,14] and the association between HIV-specific CD8+ proliferative responses and long term non progressor status [15]. Initiation of HAART in the chronic phase of infection generally results in a decline in the breadth and magnitude of the HIV-specific responses in association with viral load control [16,17].

In order to boost HIV-specific immunity and limit exposure to antiretroviral drugs, treatment interruptions (TI) are being investigated. The rationale behind TI in HIV infection is that stopping treatment allows reemergence of autologous virus, which will boost virus specific immunity that can contribute to subsequent viral load control. In subjects who start HAART in acute HIV infection, the breadth and magnitude of HIV-specific immune responses is compromised compared with that seen later in infection [18-20]. In these individuals, TIs have been used after a period on HAART to expand HIV-specific immunity [21]. This strategy of early initiation of HAART followed by a controlled TI increased HIV-specific immunity and transiently suppressed viral replication.

TI performed in the setting of chronic infection has been largely unsuccessful in stimulating immunity that controls viremia [22-24]. For this reason, therapeutic vaccination and immunomodulatory therapies, which boost HIV-specific immunity are currently being investigated for HAART treated chronically HIV-infected patients prior to TI to determine whether they induce HIV-specific immunity that improves viral load control off therapy. The use of therapeutic HIV immunization (Remune™ – HIV-1 Immunogen) in chronic HIV infection to induce HIV-specific lymphoproliferative responses (LPR) is well documented. [25-28].

We present results on within-subject changes in HIV-specific immunity induced in HIV infected patients (n = 10) in the chronic phase of infection who underwent therapy intensification and vaccination with Remune™ before multiple rounds of TI. We observed that the magnitude and breadth of HIV-specific responses detected in IFN-γ ELISPOT and intracellular cytokine secretion assays increased from on treatment time points pre-TI#1 to pre-TI#2. However, this increase in HIV-specific immune response did not correlate with the decrease in the viral load plateau seen during TI#1 to that seen during TI#2. Although our results show that HAART intensification and Remune™ vaccination were able to reduce and sustain lower VL plateau during consecutive cycles of TI, this reduction did not correlate with increases in HIV-specific responses measured.

## Methods

### Patient population and study design

Ten healthy HIV infected adults in the chronic phase of infection were enrolled in March 2000 in this proof of concept trial. The research conformed with all ethical guidelines of the authors' institutions and with human experimentation guidelines of the US Department of Health and Human Services. All participants signed informed consent. At the time of enrollment, the 10 patients had a median age of 41 (range 36 to 51) years, had been on antiretroviral therapy for a median of 4.6 (range 1.4 to11) years and had been on HAART for a median of 2.7 (range 1.4 to 3.8) years, had HIV viral loads (VL) <50 copies/ml for a median of 2 (range 0.5 to 2.1) years, and median CD4+ T-cell counts of 385 (range 230 to 990) cells/mm^3 ^(Table 1).

**Table 1:** Study Population Characteristics

**Patient No.**	**MHC Class I**			**Before HAART Intensification**		**Before First Treatment Interruption (TI)**	
	**A**	**B**	**C**	**CD4 Cell Count (cells/mm**^3^**)**	**CD4 Cell %**	**CD4 Cell Count (cells/mm**^3^**)**	**CD4 Cell %**

14001	A2/A26	B18/B39	Cw7/Cw12	990	30	1120	33
14002	A2/A1	B60/B51	Cw3/Cw14	330	25	280	25
14003	A1/A3	B7/B8	Cw7/-	650	25	590	27
14004	A2/A3	B38/B44	Cw5/Cw12	400	19	370	22
14005	A3/A69	B35/B44	Cw12/Cw7	230	21	510	27
14006	A1/-	B8/B57	Cw6/Cw7	440	23	410	24
14007	A36/A68	B7/B53	Cw4/Cw7	370	16	320	17
14008	A1/A68	B8/B60	Cw3/Cw7	350	14	720	19
14009	A3/A29	B35/B44	Cw4/Cw16	710	37	800	38
14010	A2/A66	B7/B14	Cw7/Cw8	290	24	370	37
Median (range)				476 (230–990)	23.4 (16–37)	549 (280–1120)	25.9 (17–38)

The treatment schedule included a 6-month HAART intensification phase, during which didanosine (ddI) and hydroxyurea (HU) were added to the existing regimen for the first 5 months and granulocyte-macrophage colony-stimulating factor (GM-CSF) for the first 3 months. Remune™ (10 units of p24 antigen – 100 μg total protein, in Incomplete Freund's Adjuvant) was given at month 5 of the treatment intensification phase when HU was stopped. All 10 patients completed another month of therapy intensification with ddI and were vaccinated with Remune™ at three-month intervals until the end of study. Patients were monitored for 36 months after the first TI. Blood samples were obtained at baseline, month 3, 6, of the HAART intensification phase, and every 3 months for 36 months thereafter for virological and immunological assessments. HAART and HU were resumed if VL did not decrease to <50 000 copies within 3 months or if the CD4+ counts decreased to <200 cells/mm^3^. HAART was again interrupted when viral load was <50 HIV-1 RNA copies/ml and CD4+ counts were >200 cells/mm^3 ^measured on two occasions one month apart.

### HIV quantification

Plasma viremia was determined using the Roche Amplicor Assay (Roche Diagnostics, Mississauga, Ontario, Canada) with a detection limit of 500 HIV-1 RNA copies/ml of plasma. Samples falling below the detection limit were retested with the ultrasensitive method (Ultradirect; Roche Diagnostics) with a detection limit of 50 HIV-1 RNA copies/ml.

### HLA typing

Genomic DNA for molecular HLA typing was prepared from Epstein-Barr virus (EBV)-transformed B cell lines using the QIAamp DNA blood kit (Qiagen, Mississauga, Ontario, Canada). Each patient was typed for HLA class I alleles using 95 primer sets amplifying defined MHC class I alleles (ABC SSP Unitray; PelFreez Clinical Systems, Brown Deer, Wisconsin, USA) [29]

### Cells and Peptide Selection

Peripheral blood mononuclear cells (PBMC) were isolated from blood collected in acid citrate dextrose anticoagulant at each study visit by density gradient centrifugation (Ficoll-Paque, Pharmacia, Upsala, Sweden) and frozen in 90% fetal calf serum (GIBCO BRL Life Technologies, Burlington, Ontario, Canada), 10% dimethyl sulfoxide (DMSO, Sigma, St. Louis MO). The HIV epitopes used for PBMC stimulation were chosen from the Los Alamos HIV Molecular Immunology Database [30]. Optimal peptides of 8 to 10 aa in length restricted to the MHC class I alleles expressed by the individuals being tested were synthesized to greater than 85% purity by solid phase synthesis using F-MOC chemistry (Sheldon Biotechnology Center, Montreal, Quebec, Canada). Twenty-mer peptides corresponding to HIV Gag p55 were obtained from the National Institute of Biological Standards and Controls (Potters Bar Hertz, UK). These were organized into pools containing peptides corresponding to HIV Gag p17, p24 and p15. Each peptide in these pools was present at a final concentration of 2.0 μg/ml. In addition, MHC restricted EBV- or cytomegalovirus (CMV)-derived 8- to 10-mer optimal peptides were also synthesized as described above and used as positive peptide control stimuli.

### IFN-γ Enzyme-Linked Immunospot (ELISPOT) Assay

IFN-γ secretion by HIV-specific cells was quantified by ELISPOT assay as described [20]. Panels of MHC restricted stimulating peptides were designed for each study subject and used to screen responses at each time point tested (Table 2). Panels were composed of a median of 9 (range 6 to 11 peptides) restricted to a median of 2.5 (range 2 to 5) MHC class I alleles. In addition Gag p17, p24, and p15 peptide pools were also used as stimuli. Cells were plated at both 2 × 10^5 ^cells/well and 5 × 10^4 ^cells/well for each peptide condition. Anti-CD3 monoclonal antibody (mAb) (Research Diagnostics, Flanders, NJ) and immunodominant CMV/EBV derived peptides were used as positive control stimuli whereas medium was used as a negative control. The frequency of reactivity of anti-CD3 and EBV/CMV peptides stimuli occurring in longitudinally collected samples was used to control for between-time point variability in cell responsiveness. Results are expressed as spot-forming cells (SFCs)/10^6 ^PBMC after subtraction of negative controls. A positive response met the criteria of having at least 5 spots per well and at least 2-fold more spots than the negative control wells.

**Table 2:** List of MHC class I-restricted peptides used as stimuli

**Peptide ID**	**Sequence Location**	**Sequence**	**MHC Restriction (s)**
A1-1	p17 (71–79)	GSEELRSLY	A1
A1-2	Nef (121–128)	FPDWQNYT	A1
A1-3	Nef (184–192)	RFDSRLAFH	A1
A2-1	p17 (77–85)	SLYNTVATL	A2
A2-2	RT (309–317)	ILKEPVHGV	A2
A2-3/A3-1	Nef (190–198)	AFHHVAREL	A2, A3
A2-4	p24 (19–27)	TLNAWVKVV	A2
A2-5	RT (179–187)	VIYQYMMDL	A2
A2-6	CMV	NLVPMVATV	A2
A3-2	EBV	IVTDFSVIK	A3, A11, A6801
B7-1	p24 (47–56)	ATPQDLNTML	B7, B58
B7-2	p24 (16–24)	SPRTLNAWV	B7
B7-3/B35-1	Nef (68–77)	FPVTPQVPLR	B7, B35
B7-4	Nef (128–137)	TPGPGVRYPL	B7
B7-5	CMV	TPRVTGGGAM	B7
B7-6	EBV	RPPIFIRRL	B7
B8-1	p24 (127–135)	GEIYKRWII	B8
B8-2	Nef (90–97)	FLKEKGGL	B8
B8-3	p17 (24–31)	GGKKKYKL	B8
B8-4	RT (18–26)	GPKVKQWPL	B8
B8-5	p17 (93–101)	EIKDTKEAL	B8, B60
B8-6	EBV	FLRGRAYGL	B8
B35-2	RT (175–183)	HPDIVIYQY	B35
B35-3	gp160 (41–51)	GVPVWKEATTT	B35
B35-4/B7-7	RT (156–166)	SPAIFQSSMTK	A3, A3.1, A11, A6801, A33, B7, B35
B35-5	Nef (73–82)	QVPLRPMTYK	A3, A11, A31, B27, B35
B44-1	p24 (174–184)	AEQASQDVKNW	B44, B57, Cw4
B44-2	p24 (162–172)	RDYVDRFYKTL	B18, B2601, B44, B70
B44-3	RT (203–212)	EELRQHLLRW	B44
B44-4	RT (397–406)	TWETWWTEYW	B44
Cw7-1	gp160 (37–46)	TVYYGVPVWK	A3, A6801, A11, Cw7

### Statistical analyses

Data were analyzed by using GraphPad InStat statistical software, version 3.06 [(2003) GraphPad Software, San Diego, California, USA]. Two-tailed nonparametric Wilcoxon matched-pairs signed-ranks tests were used to assess differences in VL, the magnitude and breadth of HIV-specific responses, and the percentage of Gag p55-specific CD4+ and CD8+ T-cells between each TI. Nonparametric Spearman rank correlations were used to correlate the VL improvements with both increases in the magnitude of HIV-specific responses and changes in breadth of these responses between the 1^st ^and 2^nd ^TI. The total immune responses generated were expressed as the area under the curve (AUC) calculated from total HIV-specific responses over time for each patient. Nonparametric Spearman rank correlations were used to evaluate the correlation between the total HIV-specific immune responses and the number of days patients were able to stay off HAART. All tests for statistical significance were two-tailed and *p *values <0.05 were considered significant.

## Results

### Changes in HIV-specific immune responses

**Figure 1:**
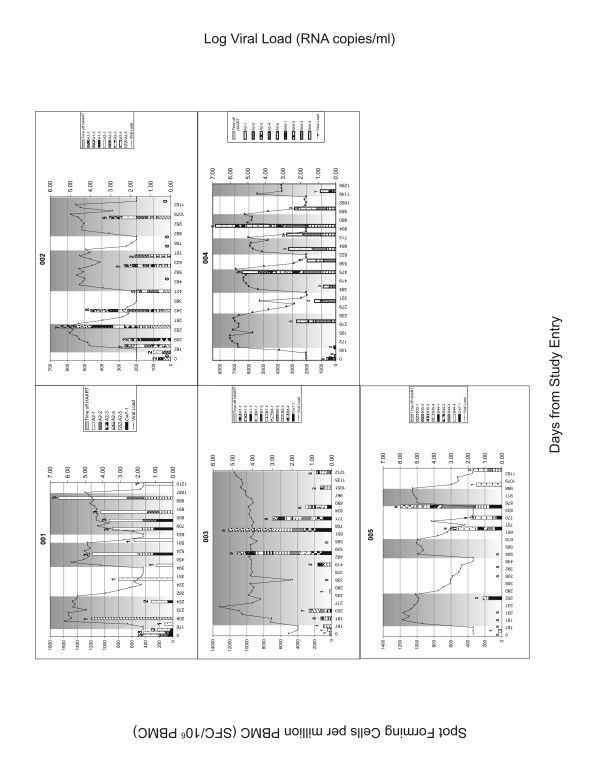
**Results of IFN-γ ELISPOT assay for patient 001 to 005**. The left y-axis shows the number of spot forming cells (SFC)/10^6 ^PBMC. Each stacked bar shows the number of SFC/10^6 ^PBMC generated to the peptide panel tested at each clinic visit. The height of the stacks in each the bar represents the number of SFC/10^6 ^PBMC induced by each positive stimulus. The height of the bar is the cumulative magnitude of the response to the peptide panel tested. The number over the bar is the number of peptides in the panel recognized at that time point. The shaded areas are the intervals off HAART. Also shown are viral load determinations at each time point keyed to the right y-axis.

**Figure 2:**
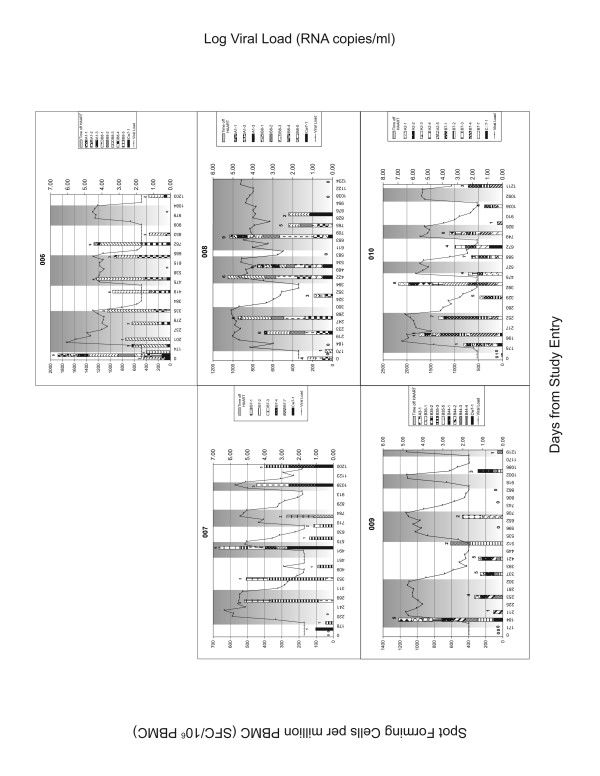
**Results of IFN-γ ELISPOT assay for patient 006 to 010**. See the legend for Figure 1.

**Figure 3:**
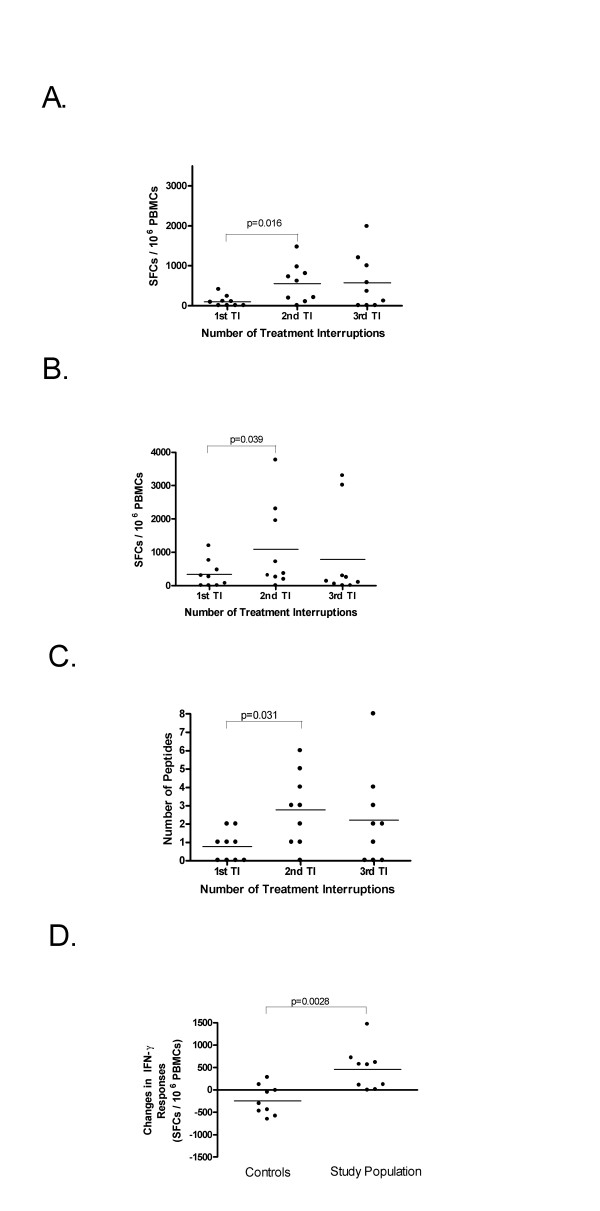
**Comparison of the magnitude and breadth of HIV-specific responses between TI#1, TI#2, and TI#3**. **A**. The magnitude of responses to peptide panels increased significantly by a mean of 457 SFC/10^6 ^PBMC from TI#1 to the TI#2 (p = 0.016), and 20 SFC/10^6 ^PBMC from the TI#2 to TI#3 (n.s.). **B**. The magnitude of responses to Gag p55 peptide pools increased by a mean of 754 SFC/10^6 ^PBMC from the TI#1 to TI#2 (p = 0.039), and decreased by a mean of 302 SFC/10^6 ^PBMC from the TI#2 to TI#3 (n.s) **C**. The breadth of responses to the HIV peptide panels used for screening increased significantly by a mean of 2.00 peptides from the TI#1 to TI#2 (p = 0.031) and decreased by a mean of 0.56 peptides from the TI#2 to TI#3 (n.s.) **D**. Comparison of the magnitude of the change in IFN-γ responses from the first to the second time point tested in continuously treated HIV-infected subjects (controls) and between TI#1 and TI#2 in the study population. The bar in each scatter plot shows the mean change in SFC/10^6 ^PBMC. The magnitude of the change differed significantly between the controls and the study population (-240 ± 331 versus +457 ± 475 SFC/10^6 ^PBMC respectively, p = 0.0028; Mann-Whitney test); n.s.= not significant.

PBMC samples from all time points were screened for HIV-specific IFN-γ secretion using panels of optimal epitopes restricted to the MHC class I alleles of the individuals being tested. These samples were also screened by IFN-γ ELISPOT assay with Gag peptides pools corresponding to HIV Gag p55. Figure 1 and 2 shows the breadth and magnitude of the response to the optimal peptide panels used to screen each individual. The magnitude of the responses to the HIV peptide panels were compared before 1, 2 and 3 TIs at time points where subjects were on HAART in order to assess whether changes in HIV-specific responses occurred with increasing numbers of TI (Figure 3). For the peptide panel stimuli, the magnitude of the HIV response increased from 102 ± 137 SFC/10^6 ^PBMC at TI#1 to 559 ± 483 SFC/10^6 ^PBMC at TI#2 and 579 ± 688 SFC/10^6 ^PBMC at TI#3 (Figure 3A). However, the increase in the magnitude of the response to peptide panels was only statistically significant for comparisons between TI#1 and TI#2 (p = 0.016, Wilcoxon matched-pairs signed-ranks test). For Gag p55 specific responses, a significant increase in magnitude was seen from TI#1 (336 ± 409 SFC/10^6 ^PBMC) to TI#2 (1090 ± 1290 SFC/10^6 ^PBMC) (p = 0.039). No further increase in the magnitude of the HIV Gag specific response was evident from TI#2 to TI#3 (789 ± 1345 SFC/10^6 ^PBMC) (Figure 3B). The breadth of the response to the HIV peptide panels (Figure 3C) also increased significantly between TI#1 (0.78 ± 0.83 peptides) and TI#2 (2.78 ± 1.99 peptides) (p = 0.031) but did not increase further at TI#3 (2.22 ± 2.59 peptides).

To compare the fate of HIV-specific IFN-γ secretion between the study population and individuals in the chronic phase of infection on continuous HAART that suppresses viremia to undetectable levels but who do not undergo therapy intensification, vaccination or TI, nine historical controls of a similar age and absolute CD4 count to the study population were assembled. The continuously treated controls were screened with an MHC class I restricted HIV peptide panel at 2 on-HAART time points separated by a time interval similar to that between pre-TI#1 and pre-TI#2 time points in the study population (p = 0.45; Mann-Whitney test). The size of the peptide panels used for both the study population and the controls was similar. The magnitude of the IFN-γ responses in continuously treated controls to the peptide panels tested did not change significantly from the first to the second time point tested (data not shown). Furthermore, comparison of the magnitude of the change in IFN-γ responses from the first to the second time point differed significantly in these two populations (-240 ± 331 versus +457 ± 475 SFC/10^6 ^PBMC in the controls and the study population, respectively, p = 0.0028; Mann-Whitney test) (Figure 3D).

In order to determine whether changes in HIV-specific immunity occurred in the CD4+ or CD8+ cell compartments (or both) we also measured percent of HIV Gag p55 specific IFN-γ secreting CD4+ and CD8+ cells by ICS as described [31]. Although changes in HIV-specific IFN-γ secretion responses detected by ICS displayed a similar trend in both compartments to that observed using the ELISPOT assay, none of these differences was statistically significant (not shown).

### Timing of appearance and magnitude of HIV-specific immune responses with control of VL after HAART is withdrawn

**Figure 4:**
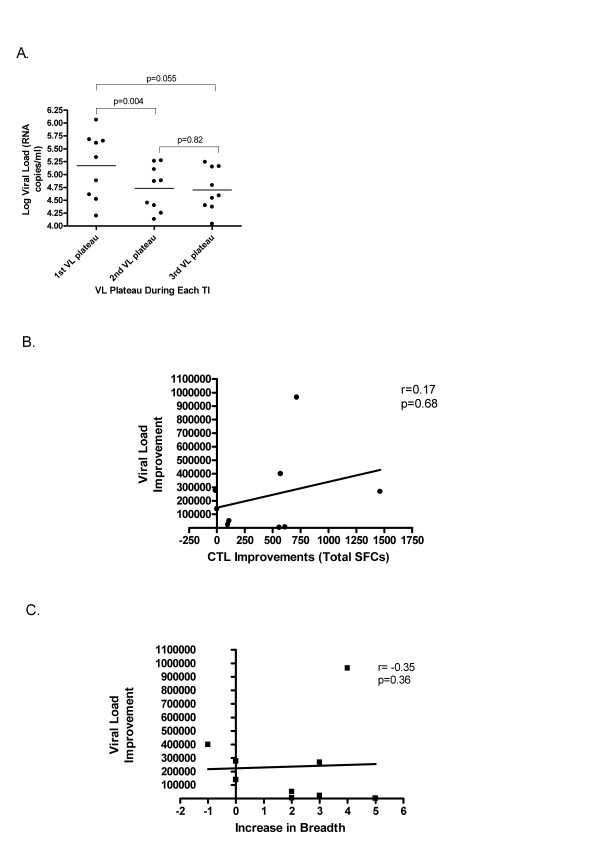
**Correlation between VL and HIV-specific responses**. A significant reduction of 0.44 log_10 _unit occurred from TI#1 VL plateau to TI#2 VL plateau (p = 0.004) and decreased 0.48 log10 units from TI#1 VL plateau to TI#3 VL plateau (p = 0.055). Despite this, no correlation was evident between VL improvement and either the increase in the magnitude or the breadth of HIV-specific immune response; VL improvement is the difference between the TI#1 and TI#2 VL plateau; increase in the magnitude is the difference in SFCs between TI#1 and TI#2; increase in breadth is the difference in the number of epitopes recognized between TI#1 and TI#2.

The VL plateau decreased 0.44 log_10 _units from that seen at TI#1 to TI#2 (p = 0.004, Wilcoxon matched-pairs signed-ranks test). The average VL decreased 0.48 log_10 _units from TI#1 to TI#3 (p = 0.055) (Figure 4A). Despite this, no correlation was evident between VL decrease with either the increase in the magnitude or the breadth of HIV-specific immune response to HLA-restricted optimal peptide panel (Figures 4B and 4C) and Gag p55 peptide pools (data not shown); VL decrease is defined as the difference between TI#1 and TI#2 VL plateaus; increase in the magnitude of HIV-specific immune responses is the difference in number of SFC/10^6 ^PBMC between TI#1 and TI#2 to the peptide panel; increase in breadth of HIV-specific immune responses is the difference in the number of epitopes recognized between TI#1 and TI#2.

The participants in this trial spent an average of 50.4% of the 36 months they were followed after stopping therapy for the first time off HAART. We therefore investigated whether there was a correlation between the percentage of time off HAART and the total HIV-specific immune responses to either the peptide panel tested or pools of peptides corresponding to HIV Gag p55. No significant association between these parameters was observed (not shown)

## Discussion

This report presents results on changes in HIV-specific immune responses in 10 subjects in the chronic phase of infection with undetectable HIV VL on HAART at study entry. All underwent 6 months of therapy intensification and received an initial dose of the therapeutic vaccine Remune™ before stopping HAART and all of them received Remune™ every 3 months for a total of 11 doses. Treatment was restarted if rebound VL did not decrease to <50 000 copies within 3 months or if the CD4+ counts decreased to <200 cells/μl during TI. HAART was again interrupted when viral load was <50 HIV-1 RNA copies/ml and CD4+ counts were >200 cells/mm^3 ^on two occasions one month apart.

We found that the average VL plateau decreased significantly with TI#1 to TI#2. Although both magnitude of breadth of immune responses to the screening peptide panel and Gag p55 peptide pools increased significantly from TI#1 to TI#2, no correlation between changes in VL and changes in immune response were detected. Patients were able stay off HAART for 50.4% of the time over 36 months of follow up. No correlation between the percentage of days off HAART and the immune responses generated was detected.

Subject 14003 was able to maintain viral load to below 40 000 copies/ml after one TI and remained off therapy for the reminder of the study and was not included in the analysis (mean VL of 27 288 copies/ml over 968 days). The VL in subject 14008 remained below 42 000 copies/ml of plasma (mean VL of 13 937 copies/ml over 637 days) after 2 TIs. This individual was included in comparisons between TI#1 and TI#2, but no data was available for this individual for TI#3. It should be noted that the absence of statistical significance between the comparisons of breadth and magnitude of HIV-specific immune responses may be related to the small size of comparison groups.

The increase in the breadth and magnitude of IFN-γ responses to the peptide panel tested from the time point prior to TI#1 to the time point prior to TI#2 differs from the fate of these parameters for HIV-specific responses observed in chronically infected subjects on continuous HAART that suppresses VL to below 50 copies/ml of plasma. First, the magnitude of the IFN-γ responses in continuously treated controls did not change significantly from the first to second time tested and the change in magnitude of IFN-γ responses from the first to the second time point differed significantly in these two populations. This supports the conclusion that the study population interventions including treatment intensification, vaccination and TI led to expansion of HIV-specific immunity.

Several factors may account for the lack of correlation between the increase in the magnitude and breadth of HIV-specific immune responses measured by IFN-γ ELISPOT and VL decrease from TI#1 to TI#2. First, the use of optimal peptide panels and Gag peptide pools corresponding to reference strain HIV isolates rather than autologous sequences may underestimate the true extent of HIV specific immunity [32]. Although the same set of stimuli were used to assess HIV-specific IFN-γ secretion at all time points, it is possible that the accumulation of viral sequences changes no longer recognized by HIV-specific cells with time reduces the correlation between this function of HIV-specific cells and VL control.

Second, the cytolytic activity of CD8+ T-cells is believed to be important in controlling the viral burden in HIV infection. Since IFN-γ secretion has been shown to be a surrogate for the level of CD8+ T-cell effector activity, IFN-γ ELISPOT and ICS are the standard techniques used to screen for antigen specific CTL [33]. However, recent studies have shown that lysosomal-associated membrane protein-1 (LAMP-1 or CD107a) expression on the cell surface could be a better marker for CD8+ T-cell cytolysis. CD107a has been shown to be upregulated following antigenic stimulation coupled with degranulation and the release of perforin [34,35]. Moreover, studies in chronic viral infection in murine models have shown that there is a hierarchical exhaustion of CD8+ T-cell functions. Virus-specific memory CD8+ T-cells progressively loose their functional capabilities in response to viral antigen recognition starting with the inability to secrete interleukin-2 (IL-2), and reduced proliferative and lytic activity. Next, the ability to secrete tumor-necrosis factor alpha (TNF-α) wanes [36]. IFN-γ secretion is the CD8+ T cell function most resistant to exhaustion. Therefore, the measurement of HIV-specific IFN-γ-secreting CD8+ T-cells might reflect an incomplete picture of HIV-specific immune responses best associated with suppression of viral replication.

As well, recent reports have shown that the breadth and magnitude of HIV-specific IFN-γ responses to all expressed HIV genes do not correlate with either VL or with rate of CD4+ T cell decline [37,38]. While it is fairly well established that HIV-specific CD8+ cells do mediate anti-viral activity, it may be that other functions of these cells correlate better with control of HIV replication than IFN-γ secretion. Studies with HIV infected long-term nonprogressors (LTNPs) showed that they have elevated HIV-specific proliferative capacity coupled to increased perforin expression when compared to HIV infected disease progressors [15]. Moreover, LTNPs possess an enhanced CD8 T-cell functional profile compared with progressors including maintenance of polyfunctional responses such as TNF-α and IL-2 secretion in addition to other functions [39]. Furthermore, aviremic patients treated during primary infection have increased HIV proliferative capacity as well as ability to maintain an HIV-specific IL-2-secreting CD4+ T-cell population when compared to viremic patients [40]. These studies suggest that it is the quality (HIV-specific IL-2 secretion and proliferation in particular), rather than the quantity of HIV-specific responses that may be better immune correlates of viral control.

## Conclusion

In summary our study showed that HAART intensification with GM-CSF, ddI and HU followed by Remune™ vaccination augmented HIV-specific IFN-γ secretion from TI#1 to T1#2 with a corresponding significant decrease in VL. However, no correlation could be established between these two phenomena. Patients were able to stay off HAART for 50.4% of the period of the study. TIs are an important part of the clinical management of HIV infected subjects because of the potential cost saving, reversion of drug-resistant virus to drug sensitive variants, and patients' request for a break from their medications. Therefore, the immunological and virological benefits observed in this proof of concept study warrant further studies with a larger patient population to identify potential protective HIV-specific immune responses induced by this therapeutic strategy of TI in combination with Remune™ vaccination. In addition, recent studies with Remune vaccination in chronic HIV-infected patients showed an induction of polyfunctional HIV-specific CD8+ T-cells with increased proliferative capacity, IL-2, MIP-1β, IFN-γ, and TNF-α secretion [41]. Thus, future immune monitoring for T-cell responses vaccine trials should include not only IFN-γ secretion, but also polychromatic flow cytometry to assess proliferation, degranulation, other cytokine and chemokine secretion as well.

## Competing interests

The author(s) declare that they have no competing interests.

## Authors' contributions

KHH was involved in data acquisition, data analysis, and drafted the manuscript. MPB and FC carried out data acquisition. ML participated in the design of the study and data analysis. DZ contributed to the study design. LC was involved in the design and coordination of the study. ET conceived of the study and edited the manuscript. NFB contributed to the study design, participated in data analysis, and critically revised the manuscript for important intellectual content. All authors read and approved the final manuscript.
